# Epoxy Nanocomposites Reinforced with Functionalized Carbon Nanotubes

**DOI:** 10.3390/polym12081816

**Published:** 2020-08-13

**Authors:** Anton Mostovoy, Andrey Yakovlev, Vitaly Tseluikin, Marina Lopukhova

**Affiliations:** 1 Laboratory “Modern Methods of Research of Functional Materials and Systems”, Yuri Gagarin State Technical University of Saratov, Polytechnichskaya St., 77, 410054 Saratov, Russia; 2 Department “Chemistry and Chemical Technology of Materials”, Yuri Gagarin State Technical University of Saratov, Polytechnichskaya St., 77, 410054 Saratov, Russia; aw_71@mail.ru; 3 Department “Technologies and Equipment for Chemical, Oil and Gas and Food Industries”, Yuri Gagarin State Technical University of Saratov, Polytechnichskaya St., 77, 410054 Saratov, Russia; tseluikin@mail.ru; 4 Department “Economics and Humanitarian Sciences”, Yuri Gagarin State Technical University of Saratov, Polytechnichskaya St., 77, 410054 Saratov, Russia; mlopuhova@yandex.ru

**Keywords:** polymer composite, epoxy oligomer, modification, plasticizer, structuring agent, MWCNTs, γ-aminopropyltriethoxysilane

## Abstract

In this article, amino functionalized multiwalled carbon nanotubes (MWCNTs) were prepared by chemical modification of the surface of a MWCNTs using γ-aminopropyltriethoxysilane (APTES) and dispersed into the epoxy composition. The modifying agent (APTES) was directly deposited on the MWCNTs surfaces. For the functionalization of MWCNTs, was used not the APTES concentrate, as it had been described in previous works, but its freshly prepared aqueous solution. Properties of APTES-treated MWCNTs were characterized by transmission electron microscope (TEM), Raman spectroscopy and FT-IR. The results showed that the functionalization and chemical compatibility of APTES-treated MWCNTs with epoxy composition provides their best dispersion in the epoxy composition, had important influence on curing behavior, structure and physicochemical properties of the epoxy composites plasticized with trichloroethyl phosphate. The results showed that the functionalization and chemical compatibility of APTES-treated MWCNTs with epoxy composition provides increased of physicomechanical properties of epoxy composites: bending stress increases by 194% and bending modulus increases by 137%, the tensile strength increases by 108% and the tensile elastic modulus increases by 52%, impact strength increases by 300%, in comparison with plasticized epoxy composite that does not contain MWCNTs.

## 1. Introduction

Research in the field of polymer modification with functional additives is widely used to develop polymer composites with a given set of consumer characteristics [1,2,3]. So to give polymer composites and products made from them improved functional characteristics, various nanomodifiers are used, for example, oxidized graphite, carbon nanotubes (CNTs) [4], fullerenes [5], graphene nanoplatelets [6,7], natural clay minerals [8,9] or metal oxides [10,11].

Currently, nanocomposites are often used in various industries, due to their unique physicomechanical characteristics, which are due to the fact that nanodispersed particles are not stress concentrators in polymer composites [4,12,13,14,15].

Taking into account excellent physical and mechanical properties, as well as high aspect ratio of carbon nanotubes (CNTs), they are believed to be a promising reinforcement phase for polymer composites among inorganic nanofillers [14]. Analysis of scientific literature was shows that often when CNTs were introduced into epoxy composites, there was no significant increase in the strength of epoxy composites, and sometimes this even was leads to its decrease, which is associated with poor adhesion between CNTs and the epoxy matrix and with the fact that CNTs are energetically more favorable to aggregate with each other [16,17,18,19,20].

To achieve the maximum effect from the addition of CNTs into polymer composites, the surface of CNTs is functionalized by various methods, such as fluorination [21], silanization [16], carboxylation and amidation [17,18]. These studies have shown that, after the introduction of functionalized CNTs, polymer composites have better thermal, electrical and mechanical properties than composites containing pristine CNTs.

In paper [22], a surface-sizing technique was offered to take full advantage of multi-walled carbon nanotubes (MWCNTs) and epoxy resins. Two surface-sizing treated MWCNTs were obtained through a ball-milling treatment of amino-functionalized MWCNTs (MWCNT-NH_2_) with *n*-butyl glycidylether (BuGE) and benzyl glycidylether (BeGE). The results showed that the functionalization of MWCNTs provides their best dispersion in the epoxy composition, had important influence on structure, physicochemical and mechanical properties: MWCNT-BeGE/epoxy nanocomposites exhibited increasing flexural strength and modulus of 22.9% and 37.8%, respectively compared with the neat epoxy.

In most modern studies, the influence of functionalized CNTs on the processes of structure formation during the curing the epoxy composites is noted. Choi et al. [23] found that amino-functionalized multiwalled carbon nanotubes (MWCNTs) accelerated the curing process of epoxy nanocomposites, and in paper [24] authors proved an increase in thermal effects during the curing of epoxy nanocomposites that contained amino-functionalized MWCNTs. The calculated values of the curing heat of epoxy composites containing amino-functionalized MWCNTs were higher than for systems with unmodified MWCNTs and even higher compared to epoxy composites not containing MWCNTs.

Besides, the gain effects from the addition of various amino-functionalized CNTs into epoxy nanocomposites were analyzed, and their positive effect on the operational characteristics of polymer composites was proved [16,17,18].

However, the amino groups on the surface of CNTs, as reactive chemical functional groups, can have an effect on CNTs dispersion, the curing reaction, and interfacial interaction in epoxy composites [23,24]. Since various amino-functionalized CNTs are diverse in reactivity, compatibility and dispersion stability, they will have different effect in the same epoxy matrix. At present, there is no complete understanding on the applicability of CNTs functionalization in the epoxy matrix with different composition.

In this article, amino-functionalized MWCNTs were prepared by chemical modification of the surface of a MWCNTs using γ-aminopropyltriethoxysilane (APTES) and dispersed into the epoxy composition. The novelty of this work lies in a new approach to the modification of MWCNTs. It is proposed to use an aqueous solution of the modifier (APTES), because the use of a concentrate of APTES is inconvenient due to the fact that a small amount of it is very difficult to distribute on MWCNTs, which are characterized by a high specific surface area. The use of an aqueous solution avoids this difficulty and uniformly to applies the modifier to the MWCNTs surface. In addition, researchers often recommended that the modifier be added directly to the epoxy composition [25,26], which also made it difficult to uniformly modify MWCNTs, therefore, in this work, we propose direct processing of MWCNTs with a modifier solution using ultrasound, which ensures a good distribution of the modifier over the MWCNTs surface. In addition, we deliberately blocked the reactive amino group of the modifier (pH of the mixture was adjusted to 5 with the introduction of acetic acid), thus we ensured that the interaction of the modifier with MWCNTs proceeded through the hydroxyl group, which was proved by IR spectroscopy. The interaction of modified MWCNTs with the epoxy oligomer proceeded at the retained amino group of the modifier, which was also proved by IR spectroscopy.

Properties of APTES-treated MWCNTs were characterized by transmission electron microscope (TEM), Raman spectroscopy, and FT-IR. The results showed that the functionalization and chemical compatibility of APTES-treated MWCNTs with epoxy composition provides their best dispersion in the epoxy composition, had important influence on curing behavior, structure, physicochemical and mechanical properties of the epoxy composites plasticized with trichloroethyl phosphate. This work shows an important reference value for modification, optimizing, designing of epoxy compositions for production of high performance MWCNTs/epoxy nanocomposites.

## 2. Materials and Methods

**Figure 1:**
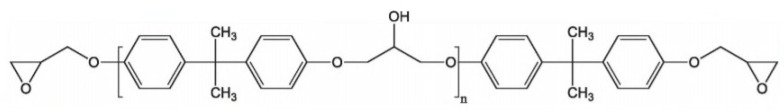
Chemical structures of epoxy resin.

Epoxy resin ED-20 (GOST 10587-93) manufactured by CHIMEX Limited (St. Petersburg, Russia) was used as a polymer matrix. Its chemical structures is shown in Figure 1.

The qualitative characteristics of ED-20 are presented in Table 1.

Polyethylene polyamine (PEPA) (TS 6-02-594-85) manufactured by CHIMEX Limited (Russia) was used as a hardener of an epoxy compositions. Its chemical formula: H_2_ N(–CH_2_CH_2_NH–)_n_H_2_; *n* = 1–4. The qualitative characteristics of PEPA are presented in Table 2.

To plasticize epoxy composites and making them flame-resistant, we used tri-2-chloroethyl ether of orthophosphoric acid (TCEP) with purity of 95.99%, which is an orthophosphoric acid and ethylene chlorohydrin ester, manufactured by PJSC “Khimprom” (Novocheboksarsk, Russia).

The choice of TCEP is due to the presence of combustion inhibitors-phosphorus and chlorine, which reduce the flammability of the epoxy composite [27,28]. The qualitative characteristics of TCEP are presented in Table 3.

Multi-walled carboxylated carbon nanotubes (MWCNTs) manufactured by "Nanotechcenter, Ltd." (Tambov, Russia) were used as a nanostructural additive. The qualitative characteristics of MWCNTs are presented in Table 4.

The surface of MWCNTs was functionalized with a γ-aminopropyltriethoxysilane (APTES) coupling agent, manufactured by the Penta-91 company (Moscow, Russia). For this purpose, 0.5 g of MWCNTs was dispersed in 100 mL of the H_2_O–APTES (95–5) solution with an ultrasonic homogenizer for 10 min. The suspension was kept under reflux at 80 °C under constant low-speed stirring at 100 rpm for 12 h. The pH of the mixture was adjusted to 5. We selected an acidic media (CH_3_COOH) in order to increase the level of silanol formation and to decrease self-condensation reactions between the hydrolyzed silanol groups. To remove the remaining silane compound around the MWCNTs particles the resulting suspension was centrifuged and washed twice with H_2_O. Then, the product was dried in the laboratory oven [29].

**Figure 2:**
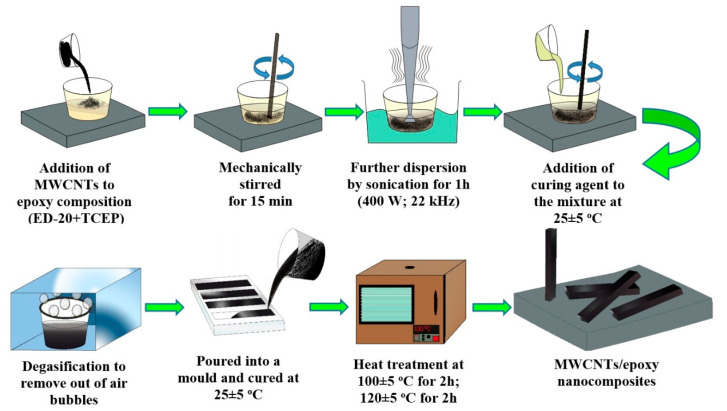
Schematic illustration for the preparation process of MWCNTs/epoxy nanocomposites.

Previously, we determined experimentally the ratio of epoxy oligomer, plasticizer and hardener: 100 parts by mass of ED-20, 40 parts by mass of TCEP and 15 parts by mass of PEPA [27,28]. MWCNTs were added as a modifying agent (0.01–0.50 parts by mass) into a plasticized epoxy composition. Ultrasonic treatment of the composition was used to increase the uniformity of distribution and hinder the aggregation of MWCNTs particles, as well as the activation of its surface and binder. The parameters of the ultrasound exposure were: frequency −22 ± 2 kHz, power −400 W, duration −60 min [30]. Before curing the mixture was degassed under vacuum at 25 ± 5 °C for 30 min. The preparation process of MWCNTs/epoxy nanocomposites was shown in Figure 2.

Bending stress and flexural modulus was determined according to ISO 178: 2010, strength and modulus of tensile elasticity was determined according to ISO 527-2: 2012, impact strength was determined according to ISO 179-1: 2010, surface morphology of the samples was studied using a Tescan VEGA 3 SBH scanning electron microscope (Brno, Czech Republic), FT-IR spectroscopy was carried out using the Shimadzu IRTracer-100 (Tokyo, Japan), Raman spectra were measured by DXR Raman Microscope (Thermo scientific, Waltham, MA, USA) with laser excitation at 532 nm, the MWCNTs structure was studied by transmitting electron microscope JEOL JEM-1400 (Tokyo, Japan). The dispersion of MWCNTs was tested by dynamic light scattering method and Nicomp 380 ZLS (Particle Sizing Systems, Inc., Santa Barbara, CA, USA) was applied. Three parallel studies were done, the coefficient of variation was 7%. Nicomp 380 ZLS is a fully automatic particle size and zeta potential analyzer. Measurement of the zeta potential, or the potential of the double layer, allows you to determine the strength of the interaction between the particles, which is a characteristic of the stability of the system and the ability of particles to agglomerate. Electrophoresis is used to determine the zeta potential in the Nicomp 380 ZLS analyzer. A small amount of sample is placed in a measuring cell with palladium electrodes that create an electromagnetic field. The specific surface area of the samples was determined by means of the Quantachrome Nova 2200 specific surface area and porosity analyzer (Boynton Beach, FL, USA) using the initial portion of the isotherm of physical nitrogen sorption (99.999%). The studied samples were placed in a cell preliminarily calibrated with respect to internal volume at a temperature of liquid nitrogen (about 78 K), which was degassed in vacuum to a constant mass at a given temperature (150 °C) for 3 h. After that, the cell was installed in the device and the isotherm of nitrogen adsorption was measured in the pressure range 0.03–0.3 P/Po. The specific surface of the test sample was calculated by the BET method with the Quantachrome Nova 2200 software [29].

## 3. Results and Discussion

As a modifying agent (0.01–0.50 parts by mass) pristine MWCNTs were added into plasticized epoxy composition (100ED-20 + 40TCEP + 15PEPA, parts by mass). Previous studies have shown that the addition of TCEP into epoxy composition provides not only an increase in mechanical characteristics (bending stress increases by 32% and impact strength is 2.6 times higher), but also reduces the flammability of the epoxy composite (oxygen index (OI) increases from 19% to 27% by volume) [27,28,30].

The conducted research has shown that the most rational content of pristine MWCNTs as a structuring additive is 0.1 parts by mass, which provides maximum values of physical and mechanical properties, Table 5, at the same time the bending failure stress increases by 135% and the bending elastic modulus increases by 90%, the tensile strength increases by 88% and the tensile elastic modulus increases by 43%, impact resilience increases by 225%.

To ensure the chemical interaction of the filler with the polymer matrix and to increase physicomechanical characteristics of epoxy composites based on them, the MWCNTs surface was treated with the APTES sizing additive.

**Figure 3:**
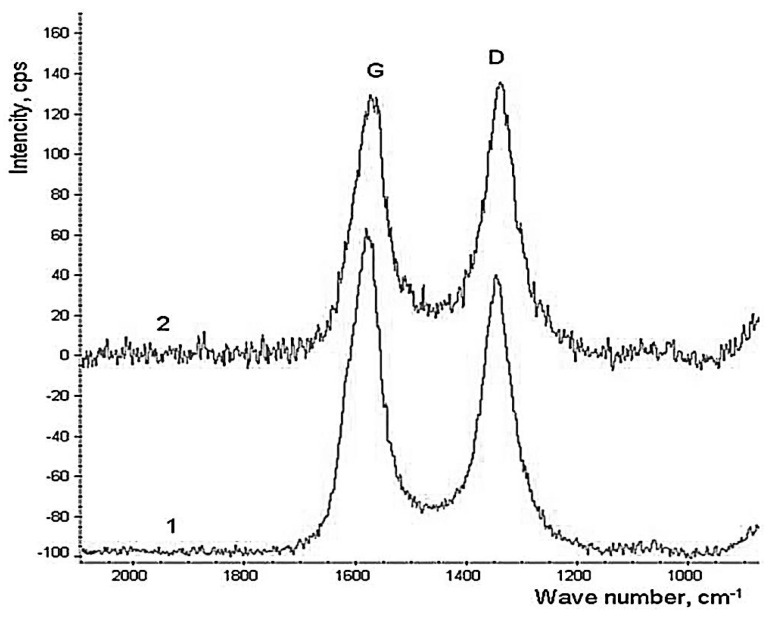
Raman spectra of (1) pristine MWCNTs and (2) APTES-treated MWCNTs.

The effect of the APTES treatment on the structure and degree of MWCNTs defectiveness was studied using Raman spectroscopy and transmission electron microscopy (TEM). Analysis of Raman spectra was shown that the R (the ratio of peak D and peak G) value of (1) is significantly smaller than (2), which infers a more regular structure of carbon atoms of (1), suggesting that the modification increased the number of defects on MWCNTs, Figure 3.

**Figure 4:**
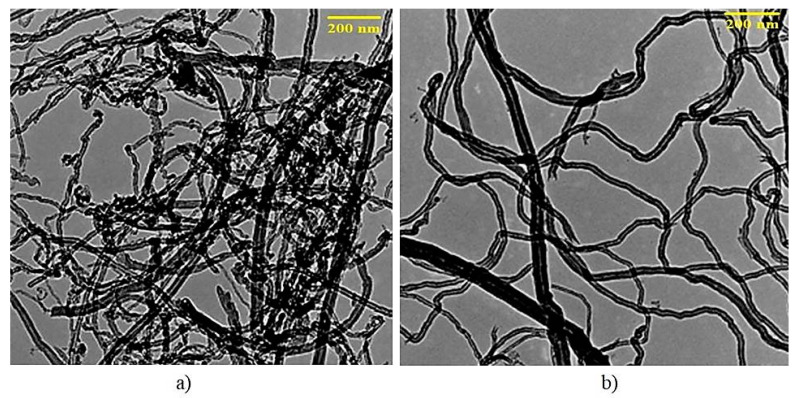
TEM photos of (**a**) pristine MWCNTs and (**b**) APTES-treated MWCNTs.

According to TEM, a decrease in aggregation and a degree of particle confusion is observed, which is confirmed by an increase in the distance between individual nanotubes due to the APTES treatment, Figure 4, as well as an increase in the specific surface of MWCNTs from 105 to 182 m^2^/g.

**Figure 5:**
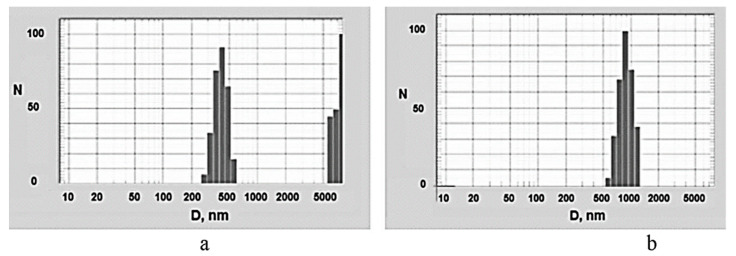
Relative amount N of agglomerates versus effective agglomerate size D (nm) for (**a**) pristine MWCNTs and (**b**) APTES-treated MWCNTs in epoxy resin diluted with isopropanol. MWCNTs concentration was equal to 0.1 wt %.

To study the dispersion of MWCNTs in an epoxy binder, a dynamic light scattering technique was used to estimate the effective particle size of the dispersed phase that would be the true size if these particles were spherical. Since MWCNTs themselves and the agglomerates formed by them are not spherical, the measurement results allow us to evaluate only the relative changes in the dispersed composition of the analyzed suspensions. As the analysis of the diagram of the suspension particles distribution by effective sizes shows, Figure 5, the suspension with pristine MWCNTs is polydisperse, Figure 5a.

The average effective size of the smallest agglomerates is 418 nm. The second peak shows an effective particle size of about 7 μm, large agglomerates slightly prevailing over smaller ones in terms of quantity. For a suspension with APTES-treated MWCNTs, the diagram of the suspension particles distribution by effective sizes is monodisperse and the average effective size of the agglomerates is in the region of 900 nm, with no larger agglomerates being observed. Thus, the APTES treatment of MWCNTs surface provides their best dispersion in the epoxy composition.

**Figure 6:**
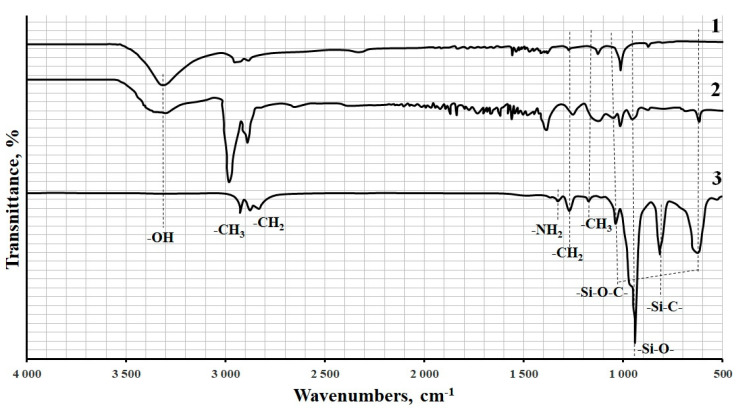
FT-IR spectroscopy: 1-pristine MWCNTs; 2-APTES-treated MWCNTs; 3-APTES.

The formation of strong bonds between MWCNTs and APTES was proved by IR Fourier spectroscopy, Figure 6. As can be seen from the spectrum of the sample after modification, vibration peaks corresponding to APTES appear; the vibration intensity of hydroxyl groups (3400–3200 cm^−1^), which are involved in the formation of the APTES layer, decreases. One can also see a broad maximum of about 1000 cm^−1^, which indicates the presence of Si–O bonds in the sample. In addition, peaks appears at 1040 cm^−1^ and 630 cm^−1^, confirming the formation of the Si–O–C bond.

The chemical interaction of the APTES functional groups and the epoxy oligomer was proved in [29].

**Figure 7:**
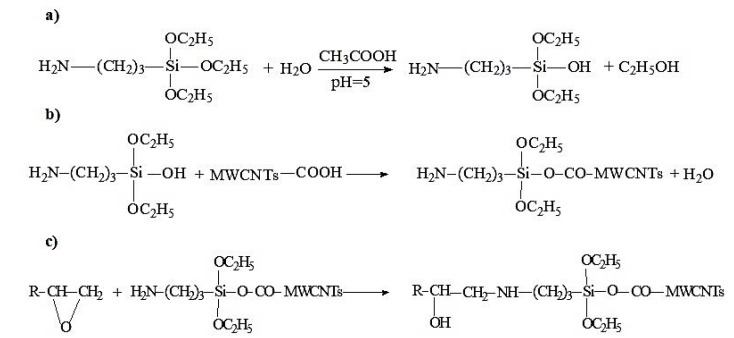
Chemistry of the interaction of epoxy oligomer ED-20, APTES and MWCNTs: (**a**) preparation of APTES solution in H_2_O; (**b**) chemical interaction of APTES and MWCNTs; (**c**) chemical interaction of APTES-treated MWCNTs with epoxy oligomer ED-20.

Based on this, it can be assumed that the reactions shown in Figure 7 take place in the system.

**Figure 8:**
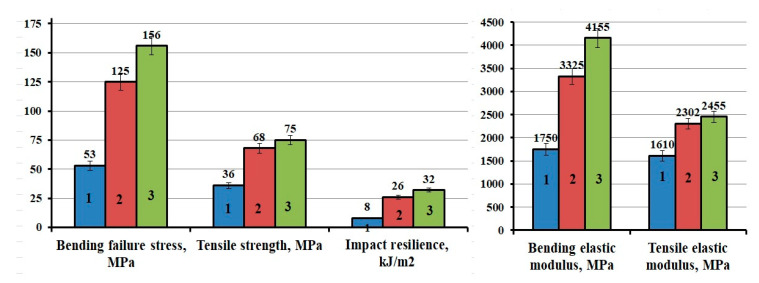
Physicomechanical characteristics of epoxy composites: 1-100ED-20 + 40TCEP+15PEPA; 2-100ED-20 + 40TCEP + 0.1MWCNTs + 15PEPA; 3–100ED-20 + 40TCEP + 0.1MWCNTs_(APTES)_ + 15PEPA.

The results showed that the functionalization and chemical compatibility of APTES-treated MWCNTs with epoxy composition provides increased of physicomechanical properties of epoxy composites: bending stress increases by 194% and bending modulus increases by 137%, the tensile strength increases by 108% and the tensile elastic modulus increases by 52%, impact strength increases by 300%, in comparison with plasticized epoxy composite that does not contain MWCNTs, Figure 8. Comparison of the obtained strengthening effects with the literature data (an increase in the physicomechanical characteristics of polymer composites by 20–40%) [4,17,26] proves the effectiveness of the MWCNTs modification method proposed in this work.

**Figure 9:**
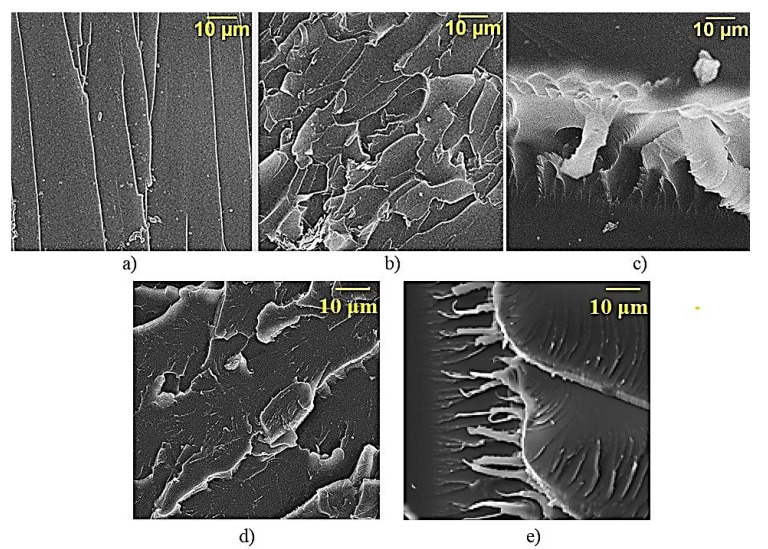
SEM of the surface of epoxy composites destruction: (**a**) unfilled composite; (**b**,**c**) composite filed with 0.1 parts by mass pristine MWCNTs; (**d**,**e**) composite filled with 0.1 parts by mass APTES-treated MWCNTs.

Figure 9a presents fractography of the destruction of epoxy composite samples without MWCNTs, which is characterized by a rather smooth fracture surface, showing a low ability to crack resistance [31]. When MWCNTs are added into the epoxy composition they affect the morphology of the matrix—there appear layered structures, Figure 9b. Along with the brittle fracture where numerous scales are formed, there are local areas in the epoxy composite, indicating the flow of material in the process of its destruction. Moreover, fibrous structures can be clearly observed in some places of plastic destruction, which are formed as a result of the intensive drawing out of the polymer matrix, Figure 9c. Considering MWCNTs as a solid phase hardener [32] we can explain the increase in plasticity of the epoxy composite. In this case, a smaller number (compared to the volume of the composition) of crosslinks is formed in the border area of MWCNTs and the epoxy composition, and, therefore, this area will have greater mobility [33].

In Figure 9d,e the destruction surface of an epoxy composite modified with amino-functionalized MWCNTs can be seen. A very large fraction of plastic destruction appears in the sample, the borders of the scales become blurred, and their size increases. An increase in the proportion of plastic deformation of the composite material confirms the hypothesis that APTES-treated MWCNTs can act as a solid-state hardener. This may be on account of the functionalization of the MWCNTs surface, which results in the formation of a dense cross-linking network around the nanotube with oligomer epoxy groups.

Nanodispersed fillers have a significant effect on the kinetic characteristics of the curing process of epoxy composites [15,30,33,34,35,36].

**Figure 10:**
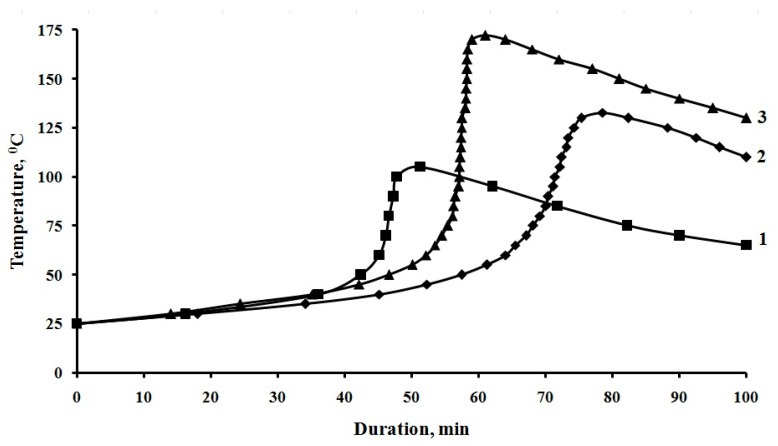
Kinetic curves of the curing process of compositions, parts by mass. 1-100ED-20 + 40TCEP + 15PEPA; 2-100ED-20 + 40TCEP + 0.1MWCNTs + 15PEPA; 3-100ED-20 + 40TCEP+0,1MWCNTs _(APTES)_+15PEPA.

The study of the curing kinetics of epoxy compounds, Figure 10, has shown that the addition of both pristine MWCNTs and APTES-treated MWCNTs affects the structure formation processes during curing of the epoxy composite.

The addition of APTES-treated MWCNTs into the epoxy composition accelerates the curing process, which can be seen in the reduction of the duration of the gelation process from 65 to 52 min, and the duration of the curing process from 78 to 61 min, with an increase in the maximum curing temperature from 132 to 172 °C. Table 6, in comparison with a composition containing pristine MWCNTs, which further confirms the participation of APTES functional groups in the curing process.

The results of measuring the coefficients of heat and thermal diffusivity are given in Table 7. The addition of both pristine MWCNTs and APTES-treated MWCNTs in the quantity from 0.05 to 0.5 parts by mass into the epoxy composite increases the thermal diffusivity by 20–42% and the thermal conductivity by 73–87%.

## 4. Conclusions

The conducted research has proven that it is possible to regulate directly operational properties of epoxy composites using small MWCNTs additives that allow to develop epoxy composites with high performance properties. The effectiveness of the surface modification of MWCNTs with γ-aminopropyltriethoxysilane and the formation of strong chemical bonds at the polymer matrix/filler interface have been proved, which ensured an increase in the physicomechanical characteristics of epoxy composites: bending stress increases by 194% and bending modulus increases by 137%, the tensile strength increases by 108% and the tensile elastic modulus increases by 52%, impact strength increases by 300%, in comparison with plasticized epoxy composite that does not contain MWCNTs.

The effect on the structure and structure formation processes during curing of epoxy compositions containing both pristine MWCNTs and APTES-treated MWCNTs has been established. The addition of APTES-treated MWCNTs into the epoxy composition accelerates the curing process, which can be seen in the reduction of the duration of the gelation process from 65 to 52 min, and the duration of the curing process from 78 to 61 min, with an increase in the maximum curing temperature from 132 to 172 °C in comparison with a composition containing pristine MWCNTs, which further confirms the participation of APTES functional groups in the curing process.

As a result of the studies, it has been proved that the addition of 0.05 to 0.5 parts by weight of both pristine MWCNTs and APTES-treated MWCNTs into the epoxy composite increases the thermal diffusivity by 20–42% and the thermal conductivity by 73–87%.

## Figures and Tables

**Table 1:** Properties of epoxy resin ED-20.

The Qualitative Characteristics of ED-20	Value
Content of epoxy groups (%)	20.0–22.5
Viscosity (Pa·s)	13–20
Epoxy equivalent (g/mol)	195–216
Density at 25 °C (kg/m^3^)	1166

**Table 2:** Properties of PEPA.

The Qualitative Characteristics of PEPA	Value
Molecular mass (g/mol)	230–250
Viscosity (Pa·s)	0.60–0.90
Density at 25 °C (kg/m^3)^	1020
Amine number (mg KOH/g)	1250
Nitrogen content (% by weight)	30.0

**Table 3:** Properties of TCEP.

The Qualitative Characteristics of TCEP	Value
Molecular mass (g/mol)	285
Viscosity (Pa·s)	0.031–0.036
Density at 25 °C (kg/m^3^)	1420–1433
Chlorine content (%) by weight	36.3–37.5
Phosphorus content (%) by weight	10.3–11.3
Boiling temperature (°C)	330

**Table 4:** Properties of MWCNTs.

The Qualitative Characteristics of MWCNTs	Value
Internal diameter (nm)	4–8
External diameter (nm)	8–15
Length (μm)	≥2
Total amount of admixtures after purification (%)	≤1
Specific surface (m^2^/g)	105

**Table 5:** Properties of epoxy composites.

Composition, Parts by Mass, Cured by 15 Parts by Mass of PEPA	G_ben_, MPa	E_ben_, MPa	G_ten_, MPa	E_ten_, MPa	a_im_, kJ/m^2^
100ED-20 + 40TCEP	53 ± 2.6	1750 ± 70	36 ± 2,1	1610 ± 65	8 ± 0.4
100ED-20+40TCEP + 0.01MWCNTs	115 ± 3.4	2522 ± 100	59 ± 3.5	2131 ± 85	20 ± 1.0
100ED-20+40TCEP + 0.05MWCNTs	120 ± 3.6	2881 ± 115	62 ± 3.7	2210 ± 88	23 ± 1.1
100ED-20+40TCEP + 0.1MWCNTs	125 ± 3.8	3325 ± 130	68 ± 4.1	2302 ± 92	26 ± 1.3
100ED-20+40TCEP + 0.5MWCNTs	98 ± 2.9	4676 ± 187	38 ± 2.3	2474 ± 98	21 ± 1.1
100ED-20+40TCEP + 0.05MWCNTs_(APTES)_	138 ± 4.2	3313 ± 125	65 ± 3.8	2320 ± 92	25 ± 1.2
100ED-20+40TCEP + 0.1MWCNTs_(APTES)_	156 ± 4.7	4155 ± 160	75 ± 4.3	2455 ± 98	32 ± 1.6
100ED-20+40TCEP + 0.5MWCNTs_(APTES)_	120 ± 3.6	5194 ± 205	46 ± 2.8	2749 ± 110	26 ± 1.3

G_ben_-bending stress; E_ben_-modulus of elasticity in bending; G_ten_-tensile strength; E_ten_ is the tensile modulus of elasticity; a_im_-impact strength.

**Table 6:** Values of the curing process of epoxy composites.

Composition, Parts by Mass, Cured by 15 Parts by Mass of PEPA	τ_gel_, min	τ_cur_, min	T_max_, °C
100ED-20+40TCEP	45	53	105
100ED-20+40TCEP+0.1MWCNTs	65	78	132
100ED-20+40 TCEP+0.1MWCNTs _(APTES)_	52	61	172

τ_gel_ is the duration of gelation process, τ_cur_ is the duration of curing, T_max_ is the maximum temperature of the self-heating of the sample during curing.

**Table 7:** Thermal diffusivity and heat conductivity of composites filled with pristine MWCNTs and APTES-treated MWCNTs.

Composition, Parts by Mass, Cured by 15 Parts by Mass of PEPA	Coefficient of Heat Conductivity, W/(m·K)	Coefficient of Thermal Diffusivity, 10^−8^ m^2^/s
100ED-20+40TCEP	0.123 ± 0.004	7.61 ± 0.26
100ED-20+40TCEP+0.05MWCNTs	0.213 ± 0.006	9.14 ± 0.28
100ED-20+40TCEP+0.1MWCNTs	0.215 ± 0.007	9.45 ± 0.28
100ED-20+40TCEP+0.5MWCNTs	0.218 ± 0.007	10.15 ± 0.30
100ED-20+40TCEP+0.05MWCNTs_(APTES)_	0.221 ± 0.007	9.22 ± 0.28
100ED-20+40TCEP+0.1MWCNTs_(APTES)_	0.224 ± 0.007	9.95 ± 0.29
100ED-20+40TCEP+0.5MWCNTs_(APTES)_	0.230 ± 0.008	10.81 ± 0.32
